# Effect of HIV-1 Subtypes on Disease Progression in Rural Uganda: A Prospective Clinical Cohort Study

**DOI:** 10.1371/journal.pone.0071768

**Published:** 2013-08-12

**Authors:** Deogratius Ssemwanga, Rebecca N. Nsubuga, Billy N. Mayanja, Frederick Lyagoba, Brian Magambo, Dave Yirrell, Lieve Van der Paal, Heiner Grosskurth, Pontiano Kaleebu

**Affiliations:** 1 Medical Research Council/Uganda Virus Research Institute Uganda Research Unit on AIDS, Entebbe, Uganda; 2 Department of Medical Microbiology, Ninewells Hospital, Dundee, United Kingdom; 3 International Rescue Committee, Dar es Salaam, Tanzania; 4 London School of Hygiene and Tropical Medicine, London, United Kingdom; The University of Hong Kong, China

## Abstract

**Objective:**

We examined the association of HIV-1 subtypes with disease progression based on three viral gene regions.

**Design:**

A prospective HIV-1 clinical cohort study in rural Uganda.

**Methods:**

Partial *gag*, *env* and *pol* genes were sequenced. Cox proportional hazard regression modelling was used to estimate adjusted hazard ratios (aHRs) of progression to: CD4≤250, AIDS onset and death, adjusted for sex, age and CD4 count at enrolment.

**Results:**

Between 1990 and 2010, 292 incident cases were subtyped: 25% had subtype A, 45% had D, 26% had A/D recombinants, 1% had C and 4% were other recombinant forms. Of the 278 incident cases included in the disease progression analysis, 62% progressed to CD4≤250, 32% to AIDS, and 34% died with a higher proportion being among subtype D cases. The proportions of individuals progressing to the three endpoints were significantly higher among individuals infected with subtype D. Throughout the study period, individuals infected with subtype D progressed faster to CD4≤250, adjusted HR (aHR), (95% CI) = 1.72 (1.16–2.54), but this was mainly due to events in the period before antiretroviral therapy (ART) introduction, when individuals infected with subtype D significantly progressed faster to CD4≤250 than subtype A cases; aHR (95% CI) = 1.78 (1.01–3.14).

**Conclusions:**

In this population, HIV-1 subtype D was the most prevalent and was associated with faster HIV-1 disease progression than subtype A. Further studies are needed to examine the effect of HIV-1 subtypes on disease progression in the ART period and their effect on the virological and immunological ART outcomes.

## Introduction

There are two types of Human Immunodeficiency Virus (HIV); HIV-1 which has a global distribution and HIV-2 that is mainly confined to West and Central Africa [1]. HIV-1 is divided into groups M (Major), N (Non-M non-O), O (Outlier) and a new group named P (pending the identification of further human cases) recently identified in two Cameroonian patients [2], [3]. Group M is further divided into 9 genetic subtypes (A–D, F–H, J–K) with subtype A further subdivided into 4 sub-subtypes (A1, A2, A3, A4) and subtype F into 2 sub-subtypes (F1 and F2). At least 55 circulating recombinant forms (CRFs) along with a myriad of unique recombinant forms (URFs) have been identified (http://www.hiv.lanl.gov/content/sequence/HIV/CRFs/CRFs.html, June 2013). Majority of HIV-1 subtypes, CRFs and URFs are in Sub-Saharan Africa which is the worst affected by the HIV epidemic. The impact and implications of the high genetic diversity of HIV on transmission, disease progression, antiretroviral therapy (ART), vaccine design and development have been reported in a number of studies however more research is required to fully understand these implications.

One study in Uganda reported that subtype A had a significantly higher transmission rate than subtype D, partially explaining the observed increase in prevalence of subtype A in some populations [4], [5]. We have however reported no significant changes in subtype proportions in our populations over time [6], [7]. Other studies in Tanzania and Uganda have shown that subtype D is associated with faster disease progression compared to other subtypes and recombinant forms [8]–[13]. In these studies, individuals infected with subtype D had significantly higher rates of CD4+ T-cell loss and a shorter progression time to AIDS or death than other subtypes and recombinants. Explanations for these observations are not fully understood, however, a study in Uganda showed that individuals having non-AIDS clinical status were more likely to be infected with X4 subtype D viruses than X4 subtype A viruses [14]. Slow or non syncytium-inducing (NSI) viruses have been shown to use the fifth receptor for CC chemokines (CCR5) for viral entry while rapid or high syncytium-inducing (SI) viruses use the fourth receptor for CXC chemokines (CXCR4) [15], [16]. This observation could possibly explain the faster disease progression rates in subtype D infected individuals.

A study comparing HIV disease progression in ethnic Africans and Swedes found no differences in disease progression between subtype A and D, however the small sample size might have biased their results [17]. Observed differences in disease progression and transmission rates between subtypes may in part explain the changing trends in subtype distribution in some populations but migration may also contribute to the changing subtype distribution [5], [18]. In Uganda, subtype D has been associated with development of HIV dementia in individuals with advanced HIV immunosuppression and at risk of developing HIV-associated cognitive impairment [19]. In addition, HIV-1 subtypes in Uganda have also been shown to have an effect on response to antiretroviral therapy [20] where subtype D was associated with faster CD4+ T-cell decline before ART and was shown to lead to a higher rate of virological failure after ART initiation. The virological and immunological mechanisms for this observation remain unknown.

Additionally, circulating HIV subtypes in a particular population have implications on the design and development of a protective vaccine [21]–[23]. Surveillance of circulating subtypes and recombinants may therefore be important for clinical management and preparation for vaccine studies. One shortfall is that most of the earlier studies inferred HIV-1 subtype by analysis of one gene to determine the effect of HIV-1 subtypes on disease progression. In this work we report on the subtype distribution and the effect of these subtypes on disease progression in a prospective population-based clinical cohort in rural South-west Uganda by subtyping partial *env*, *gag* and *pol* genes. We further assess for any differences on the effect of subtypes on disease progression in the periods before and after the introduction of ART in this population.

## Methods

### Study Population

The study participants were HIV incident ART naive individuals aged 13 years and above. Participants were enrolled in a prospective HIV-1 clinical cohort study in rural Uganda between 1990 and 2010. The clinical cohort was established to study the natural history of HIV-1 infection [24] and is nested in a larger general population cohort (GPC) that was established to study the population dynamics of the HIV epidemic [25], [26]. HIV incident cases with estimated dates of seroconversion were identified from the GPC and invited to enrol in the clinical cohort if the interval between last negative and first positive HIV test was less than 4 years. Study participants were routinely followed up quarterly, but also attended the clinic whenever necessary. The World Health Organisation (WHO) clinical staging was done at every routine visit, both by clinicians, and through a computer-based algorithm. Vital status was ascertained every year for participants who did not attend the clinic for follow-up. Since 2004 free ART has been provided to all eligible study participants according to the Ministry of Health National ART guidelines [27], [28].

### Laboratory Methods

#### CD4 cell counts measurements

Throughout the study, CD4 cell counts measurements were done at enrolment and every 6 months for HIV positive participants not on ART. Between 1990 and 1994, CD4 cell counts were measured in an external laboratory by Flow Cytometry method while between 1995 and 2010, CD4 cell counts were measured in our laboratory using the FACSCount method (Becton Dickinson, San Jose, CA, USA).

#### Viral load measurements

Since 1996, viral loads were measured at enrolment and annually for all HIV positive participants not on ART. Prior to January 2004 we used the VERSANT RNA 3.0 (Bayer, Bayer HealthCare, NY, USA) assay (lower detection limit of 50 copies/ml). From October 2007 we used the Amplicor MONITOR 1.5 (Roche, Roche Molecular Systems, NJ, USA) assay (lower detection limit of 400 per copies/ml).

#### Sample collection and HIV-1 subtyping

At enrolment, all HIV-1 incident cases had 4 mls of blood collected in an EDTA tube. The samples were transported within 24 hours to the Medical Research Council/Uganda Virus Research Institute (MRC/UVRI) Uganda Research Unit on AIDS Basic Science laboratory in Entebbe for testing, processing and storage of plasma and packed cells. A total of 209 samples from incident cases enrolled between 1990 and 2003 were analysed as follows: Proviral DNA was extracted from whole blood using a Puregene kit (Gentra Systems Inc., North Carolina, USA), according to manufacturer’s instructions. The DNA was amplified directly (without cloning) by nested PCR in both the v3/v4 region of the *env* gene (approximately 400 bp) and a region of the *gag* gene encompassing most of both p17 and p24 (approximately 720 bp). Sequencing and phylogenetic analysis using the fastDNAml and Treecon package softwares was done as previously reported [29], [30]. Samples from 83 incident cases enrolled between 2004 and 2010 were subtyped by sequencing and phylogenetic analysis in the *pol*-IN, *gp*-41 and *gag*-p24 genes and subtypes assigned using the REGA HIV-1 subtyping tool as previously described [31], [32]. Different genomic regions were sequenced in the two study periods due to differences in the study objectives of the two periods and cost of the assays.

### Statistical Analysis

Study participants characteristics were examined, tabulated and the proportions of males and females were compared using the one-sided two-sample test of proportions. Comparison of means and medians of various characteristics by gender and HIV-subtype was done using the one-sided t-test and Kruskal-Wallis test respectively. Study endpoints were defined as: CD4≤250 cells/mm^3^, WHO clinical stage 4 (AIDS) and death before (1990–2003) and after ART introduction (2004–2010). Participants were eligible for inclusion in analysis as events if they had not accessed ART prior to reaching the end point. Those who accessed ART prior to the end point where censored at the ART start date. For the progression to CD4≤250 outcome, we also considered individuals who started ART prior to reaching CD4≤250 as events for this outcome with ART start date as the event time.

Cox proportional hazard regression models were used to estimate hazard ratios (HRs) of disease progression to each endpoint for the HIV-1 subtypes adjusting for sex, age at seroconversion and baseline CD4 cell count. As a standard practice, baseline viral load was fitted as a logarithm-transformed variable. Baseline CD4 cell count depicted non-linear relationship with the various outcomes and was therefore fitted as a square root, square and cube transformed variable. Subsequent viral load and CD4 cell counts were not included in the model since these are proxy for disease progression.

### Ethics Statement

The study was approved by the Uganda Virus Research Institute Institutional Review Board and the Uganda National Council for Science and Technology. For study participation, specimens collection and subsequent analysis, at enrolment all participants aged 18 years and above gave signed or thumb-printed written informed consent, participants aged 13–17 years gave formal assent and in addition a parent or guardian gave signed or thumb-printed written informed consent. Confidentiality procedures were adhered to throughout the study.

## Results

### Participants’ Enrolment Characteristics

Between 1990 and 2010, 590 HIV incident cases were invited to enrol in the clinical cohort, of whom 382 (65%) were enrolled. Of those enrolled, 292 (76%) met the study inclusion criteria. We report findings from the 292 HIV-1 subtyped cases, of which 57% were female. At HIV seroconversion, first HIV positive test and enrolment in the cohort, males were significantly older than females (p<0.01). At enrolment, there were no significant gender differences in mean viral load and median CD4 cell count (Table 1).

**Table 1:** Characteristics of HIV-1 incident cases in the cohort: 1990–2010.

Characteristic	Males	Females	Total	P-value
Total	127 (43%)	165 (57%)	292 (100)	0.01*
**Estimated age (years)**				
At HIV sero-conversion				
Mean (SD)	35 (12.5)	30 (12.4)	32 (12.6)	<0.01+
Median (IQR)	31 (25–41)	28 (21–35)	30 (23–37)	<0.01&
Range	18–79	13–72	13–79	
**At first positive HIV test result**				
Mean (SD)	35 (12.4)	31 (12.2)	33 (12.5)	<0.01+
Median (IQR)	31 (26–41)	28 (21–35)	30 (23–38)	<0.01&
Range	18–79	14–72	14–79	
**At enrolment in cohort**				
Mean (SD)	35 (12.5)	31 (12.2)	33 (12.5)	<0.01+
Median (IQR)	31 (26–41)	28 (21–35)	30 (23–38)	<0.01&
Range	19–79	14–72	14–79	
**Virological and immunological status at enrolment**				
Mean (SD) viral load (log_10_ copies/ml)	(n = 122)	(n = 155)	(n = 277)	0.167+
	4.63 (0.92)	4.53 (0.80)	4.57 (0.85)	
Median (IQR) CD4 cell counts (cells/mm^3^)	576 (459–758)	587 (425–895)	577 (435–845)	0.65&

* one-sided two sample test of proportion.

+ one-sided t-test.

& Kruskal-Wallis test.

### HIV-1 Subtype Distribution and Baseline Virological and Immunological Status

Subtype D was the most prevalent in all the three regions of *env*, *gag* and *pol* and in the combined regions. During the pre-ART (1990–2003) and whole (1990–2010) periods, the proportion of subtype D incident cases was significantly higher than that of both subtype A and A/D recombinants (p<0.01). In the combined region, HIV-1 subtypes were similarly distributed over the whole and in the pre-ART periods, however, in the ART period there was a slight increase in subtype A cases (Table 2). The subtype and disease progression analysis was based on the 278 (95% of the subtyped) cases of subtypes A, D, and A/D recombinants. The mean baseline viral load (log10 copies/ml) was significantly different among the three subtypes A, D and A/D recombinants (one way anova p-value = 0.01). Subtype A cases had the lowest mean baseline viral load, followed by A/D recombinants and then subtype D cases. There were no significant differences in the distribution of baseline CD4 cell counts between the HIV subtypes (Table 2).

**Table 2:** HIV-1 subtype distribution in env, gag, pol and combined regions; and virological and immunological status of HIV-1 incident cases at enrolment.

HIV Subtype	Region				Combined region (subtypes A, D & A/D)			Viral load and CD4 cell counts distribution by combined regions subtypes			
	*Env*	*gag*	*pol*	combined (*env/gag/pol*) whole period	Whole period	Pre-ART	ART period	Viral load (log_10_ copies/ml)		CD4 cell counts (cells/mm^3^)	
	n (%)	n (%)	n (%)	n (%)	n(%)	n(%)	n(%)	Mean (SD); P	Median (IQR); P	Mean (SD); P	Median (IQR); P
**A**	117 (43)	101 (36)	38 (27)	72 (25)	72 (26)	48 (24)	24 (31)	4.31 (0.755)	4.36 (3.89– 4.87)	627 (302.1)	571 (432–789)
**D**	146 (53)	158 (56)	92 (65)	130 (44)	130 (47)&&	96 (48)&&	34 (44)	4.71 (0.909); p<0.01^+^	4.72 (4.21–5.34); p<0.01&	676 (337.9); p = 0.15^+^	607 (439–883); p = 0.36&
**A/D**	5 (2)	15 (5)	9 (6)	76 (26)	76 (27)	56 (28)	20 (26)	4.55 (0.846); p = 0.043^+^	4.68 (4.09–5.15); p = 0.046&	642 (337.6); p = 0.38^+^	565 (414–852); p = 0.93&
**C**	4 (1)	5 (2)	2 (2)	3 (1)							
**Recombinants (A/C, A/D/C, D/C, D/B)**	3 (1)	4(1)	-	11(4)							
**Total**	275 (100)	283 (100)	141 (100)	292 (100)	278 (100)	200 (100)	78 (100)				

&& Proportion of subtype D is greater than that of A (p<0.01) and of A/D (p<0.01).

+one-sided t-test; subtype A – baseline.

& Kruskal-Wallis test.

### Effect of HIV-1 Subtypes on Disease Progression

By December 2010, of the 278 cases, 62% had progressed to CD4 count ≤250 and 32% to AIDS, while 34% had died. Before ART introduction and over the whole study period, the proportion of cases progressing to any of the three outcomes was highest among subtype D cases followed by A/D recombinants (Table 3). During the ART period, there were only two cases valid for inclusion in the AIDS and death outcome analyses, therefore further analyses for these outcomes were not performed in this period. Subtype D cases significantly progressed faster to CD4≤250 than subtype A cases during both the pre-ART period [adjusted Hazard Ratio (aHR) = 1.78, 95% Confidence Interval (95% CI): 1.01–3.14] and the whole period [aHR = 1.72, (95% CI: 1.16–2.54)]. During the three analysis periods, females progressed faster to CD4≤250 than males, while in the pre-ART and whole study period, older age at seroconversion was significantly associated with faster progression to CD4≤250. Higher baseline CD4 cell count was protective in all the three analysis periods (Table 4, Part 1).

**Table 3:** Number (proportion (%)) of participants progressing to each outcome by HIV-1 subtype.

	Outcome: CD4≤250			Outcome: WHO-stage 4: AIDS			Outcome: Death		
Subtype	Pre-ART	ART period	Whole period	Pre-ART	ART period	Whole period	Pre-ART	ART period	Whole period
**A**	18 (19)	14+5*(24)	37 (21)	11 (18)	5+0*(19)	16 (18)	14 (19)	6+1*(32)	21 (22)
**D**	49 (52)&&	26+14*(51)	89 (51)&&	28 (46)&	13+1*(52)	42 (48)&	36 (49)&	12+0*(55)	48 (51)&&
**A/D**	28 (29)	15+4*(24)	47 (27)	22 (36)	7+1*(30)	30 (34)	23 (32)	2+1*(14)	26 (27)
**Total**	95 (100)	55+23* (100)	173 (100)	61 (100)	25+2*(100)	88 (100)	73 (100)	20+2*	95 (100)

* Number not valid for analysis in the ART period; sero-converted after ART roll out.

&& Proportion of subtype D is higher than that of A (p≤0.01) and of A/D (p<0.05).

& Proportion of subtype D is higher than that of A (p≤0.05).

**Table 4:** Cox proportion hazard regression modelling outcome: CD4≤250, WHO clinical stage 4 and death.

	Characteristic	Pre-ART Period: 1990–2003				ART period: 2004–2010*				Whole period: 1990–2010			
		Unadjusted analysis		Analysis adjusted for the other variables		Unadjusted analysis		Analysis adjusted for the other variables		Unadjusted analysis		Analysis adjusted for the other variables	
Outcome		HR (95% CI)	P	HR(95% CI)	P	HR (95% CI)	P	HR(95% CI)	P	HR (95% CI)	P	HR(95% CI)	P
**Part I: CD4≤250**	Sex (M baseline)	1.39 (0.92–2.10)	0.113	2.06 (1.33–3.19)	**0.001**	3.67 (1.09–12.39)	0.036	4.25 (1.21–14.97)	0.024	1.41 (1.04–1.91)	**0.028**	2.02 (1.45–2.82)	**<0.001**
	Age at sero-conversion	1.02 (0.999–1.04)	**0.057**	1.02 (1.01–1.04)	**0.008**	1.01 (0.98–1.05)	0.491	1.01 (0.98–1.04)	0.629	1.02 (1.00–1.03)	**0.011**	1.02 (1.01–1.04)	**0.001**
	Subtype D&	1.19 (0.69–2.05)	0.52	1.78 (1.01–3.14)	**0.048**	2.34 (0.84–6.5)	0.104	1.40 (0.48–4.14)	0.539	1.30 (0.88–1.91)	0.183	1.72 (1.16–2.54)	**0.007**
	Subtype A/D&	1.11 (0.61–2.01)	0.732	1.13 (0.61–2.10)	0.697	1.10 (0.29–4.11)	0.887	1.67 (0.42–6.54)	0.464	1.12 (0.73–1.73)	0.606	1.30 (0.83–2.04)	0.255
	Baseline CD4&&					0.46 (0.33–0.62)	**<0.001**	0.45 (0.31–0.65)	**<0.001**	0.54 (0.48–0.62)	**<0.001**	0.55 (0.47–0.64)	**<0.001**
	Square root of baseline CD4&&	0.27 (0.198–0.37)	**<0.001**	0.24 (0.18–0.34)	<0.001								
	Squared Baseline CD4&&									1.02 (1.02–1.03)	**<0.001**	1.02 (1.01–1.03)	**<0.001**
**Part 2: WHO stage** **4 (AIDS)**	Sex (M baseline)	1.27 (0.77–2.11)	0.351	1.67 (0.98–2.84)	**0.059**					0.94 (0.62–1.43)	0.767	1.06 (0.68–1.64)	0.796
	Age at sero-conversion	1.01 (0.99–1.04)	0.215	1.02 (0.99–1.04)	**0.099**					1.01 (0.99–1.03)	0.186	1.01 (0.99–1.03)	0.153
	Subtype D&	1.07 (0.53–2.16)	0.843	1.42 (0.69–2.92)	0.341					1.47 (0.83–2.62)	0.188	1.61 (0.90–2.87)	0.111
	Subtype A/D&	1.32 (0.64–2.74)	0.453	1.61 (0.76–3.41)	0.211					1.66 (0.90–3.05)	0.102	1.88 (1.01–3.48)	**0.045**
	Baseline CD4&&	0.84 (0.76–0.92)	**<0.001**	0.83 (0.76–0.91)	**<0.001**					0.94 (0.88–1.01)	0.102	0.94 (0.87–1.00)	**0.066**
**Part 3: Death**	Sex (M baseline)	1.01 (0.64–1.60)	0.966	1.62 (0.997–2.63)	**0.052**					1.06 (0.71–1.59)	0.774	1.25 (0.81–1.94)	0.317
	Age at sero-conversion	1.05 (1.03–1.07)	**<0.001**	1.05 (1.03–1.08)	**<0.001**					1.04 (1.02–1.05)	**<0.001**	1.05 (1.03–1.06)	**<0.001**
	Subtype D&	1.08 (0.58–2.01)	0.803	1.83 (0.95–3.55)	**0.072**					1.22 (0.73–2.03)	0.454	1.18 (0.69–1.99)	0.544
	Subtype A/D&	1.01 (0.52–1.97)	0.984	1.47 (0.72–2.99)	0.293					1.15 (0.65–2.05)	0.63	1.30 (0.72–2.32)	0.383
	Baseline CD4&&									0.75 (0.67–0.84)	**<0.001**	0.72 (0.64–0.81)	**<0.001**
	Squared baseline CD4&&	0.95 (0.94–0.97)	**<0.001**	0.95 (0.94–0.97)	**<0.001**								
	Cubed baseline CD4&&	1.00 (1.002–1.003)	**<0.001**	1.00 (1.002–1.004)	**<0.001**					1.001 (1.0006–1.0014)	**<0.001**	1.001 (1.0008–1.002)	**<0.001**

& With subtype A as baseline.

&& Baseline CD4 counts scaled by 100.

* Analysis for WHO stage and death outcomes not done for the ART period: there were only three events.

Over the whole study period, HIV-1 A/D recombinant cases progressed faster to AIDS than subtype A cases [aHR = 1.88, (95%CI: 1.01–3.48)]. During both the pre-ART and whole periods, higher baseline CD4 cell count offered protection against progression to AIDS at a significant adjusted rate of 17% during the pre-ART period (Table 4, Part 2). During the pre-ART period, subtype D cases had a non-significant faster progression to death than subtype A cases [aHR = 1.83, (95% CI: 0.95–3.55)]. Older age at seroconversion was significantly associated with faster progression to death during both the pre-ART and whole periods. High baseline CD4 cell count offered better survival (Table 4, part 3).

## Discussion

In this rural Ugandan population, over a two decades study period we found that individuals infected with HIV-1 subtype D, the most prevalent subtype, had a significantly faster disease progression to CD4 cell counts ≤250 cells/mm^3^ than those infected with subtype A. Although non-significant, HIV-1 subtype D cases also had a faster disease progression to AIDS and to death than subtype A cases. Compared to subtype A cases, A/D recombinants cases had a significant faster disease progression to AIDS, but the progression to CD4 cell counts ≤250 cells/mm^3^ and death was not significantly faster. Female gender was associated with a significantly faster disease progression to CD4 cell counts ≤250 cells/mm^3^ than among males. Older age at seroconversion and lower baseline CD4 cell counts were significantly associated with faster disease progression to CD4 cell counts ≤250 cells/mm^3^ and death. After the introduction of ART, the association between HIV subtype and disease progression became non-significant, though we did not have enough events to assess progression to AIDS and death during this period.

The strength of our study was the long follow-up time with regular clinical and laboratory data that allowed us to examine disease progression over a two decades period. Our study was however limited by our failure to control for host genetic factors like HLA, which might influence HIV disease progression. In addition, we did not have HIV-1 subtype data on 51% of the incident cases in this population (208 not enrolled in the cohort and 90 enrolled but not subtyped). This could have introduced a selection bias that might have affected the observed results. Though we inferred HIV-1 subtype by sequencing partial *env*, *gag* and *pol* genes, this could have possibly misclassified some real recombinants as pure subtypes. Also there may have been a possibility that some reported recombinants could have been HIV-1 dual/multiple infections (infection with more than one HIV-1 subtype or strain), which have been associated with faster disease progression [32], [33]. Therefore whole genome sequencing could have been the ideal method to infer subtype.

An earlier study did not find association between HIV subtypes and disease progression leading to a conclusion that probably determination of genetic HIV subtype had little value in routine clinical patient care. This study was however limited by a small sample size and short follow-up [17], [34]. Our findings are in conformity with a study in a neighbouring district which reported shorter survival times among individuals infected with subtype D and A/D recombinants compared to those infected with subtype A [10]. Other studies on the effect of HIV-1 subtypes on disease progression have also reported that HIV subtype D is associated with faster disease progression compared to subtype A [8]–[10], [12]–[14], [35]. In Senegal, women infected with non-A HIV subtypes were 8 times more likely to progress to AIDS than those infected with the predominant subtype A [36].

The faster rates of CD4 T-cell decline associated with subtype D and a higher probability of having an X4 virus in subtype D infections (compared to subtype A) had earlier been given as possible explanations for the faster disease progression among those infected by subtype D than subtype A [8], [14], [37]. However, a later study investigating the number of CD4+ T cells expressing CCR5 or CXCR4 found specific alterations in the abundance of CD4+/CCR5+ and CD4+/CXCR4+ T cells and in immune proteins concentrations across different HIV clades. The authors concluded that these changes were unlikely to explain the differences observed in disease progression between subtype A and D cases [38].

Although for the whole study period we found a faster disease progression to CD4 cell counts ≤250 cells/mm^3^ among individuals infected with subtype D than those infected with subtype A, this was mainly due to the observations in the period before ART introduction. In the ART period, we did not find association between HIV-1 subtype and disease progression to CD4≤250 cells/mm^3^ or AIDS, because individuals started ART before these endpoints were reached. The faster progression of subtype D cases to CD4≤250 cells/mm^3^ implies that they will initiate ART earlier than subtype A cases.

The odds of progressing to AIDS and death were not significantly higher among subtype D compared to subtype A possibly because of the lower proportion of participants progressing to these end points than that progressing to CD4 cell counts ≤250 cells/mm^3^. In Uganda, an AIDS diagnosis is an independent ART eligibility criterion, while the CD4 cell count threshold for ART eligibility has changed from equal to or less than 200 cell/mm^3^ at the start of ART roll out in 2004 to equal to or less than 250 cells/mm^3^ in 2008.

Our finding that older age at seroconversion is associated with faster disease progression is in agreement with our earlier findings [39] and with findings from the neighbouring Rakai district [11], Zambia [40] and Greece [41]. In our study, 57% of patients were female, this finding is not surprising and is supported by the WHO/UNAIDS estimates in 2010 when women constituted 59% of all HIV infected patients in sub-Saharan Africa [42]. Our finding that females had a faster disease progression to CD4 cell counts ≤250 cells/mm^3^ than males is in conformity to what has been reported from Zambia [40]. Although the biological mechanisms of the gender difference in HIV disease progression remain unclear, reports from the USA have also reported that women progressed faster and had more rapid declines in CD4 cell counts than men [43], [44]. The findings that lower baseline CD4 cell counts is associated with faster disease progression to CD4 cell counts ≤250 cells/mm^3^ and death are similar to those reported from a Ugandan seroprevalent HIV cohort [45] and a community-based cohort in Zambia [40].

### Conclusion

In this population, HIV-1 subtype D was the most prevalent and was associated with faster HIV-1 disease progression than subtype A. Further studies are needed to examine the effect of HIV subtypes on disease progression in the ART period and their effect on ART virological and immunological outcomes.

## References

[1] McCutchan FE (2006) Global epidemiology of HIV. J Med Virol 78 Suppl 1S7–S12. 16622870 10.1002/jmv.20599

[2] Plantier JC, Leoz M, Dickerson JE, De Oliveira F, Cordonnier F, et al (2009) A new human immunodeficiency virus derived from gorillas. Nat Med 15: 871–872. 19648927 10.1038/nm.2016

[3] Vallari A, Holzmayer V, Harris B, Yamaguchi J, Ngansop C, et al (2011) Confirmation of putative HIV-1 group P in Cameroon. J Virol 85: 1403–1407. 21084486 10.1128/JVI.02005-10 PMC3020498

[4] Kiwanuka N, Laeyendecker O, Quinn TC, Wawer MJ, Shepherd J, et al (2009) HIV-1 subtypes and differences in heterosexual HIV transmission among HIV-discordant couples in Rakai, Uganda. AIDS 23: 2479–2484. 19841572 10.1097/QAD.0b013e328330cc08 PMC2910931

[5] Conroy SA, Laeyendecker O, Redd AD, Collinson-Streng A, Kong X, et al (2010) Changes in the distribution of HIV type 1 subtypes D and A in Rakai District, Uganda between 1994 and 2002. AIDS Res Hum Retroviruses 26: 1087–1091. 20925575 10.1089/aid.2010.0054 PMC2965693

[6] Yirrell DL, Kaleebu P, Morgan D, Hutchinson S, Whitworth JA (2004) HIV-1 subtype dynamics over 10 years in a rural Ugandan cohort. Int J STD AIDS 15: 103–106. 15006072 10.1258/095646204322764299

[7] Kapaata A, Lyagoba F, Ssemwanga D, Magambo B, Nanyonjo M, et al.. (2012) HIV-1 Subtype Distribution Trends and Evidence of Transmission Clusters among Incident Cases in a Rural Clinical Cohort in South-west Uganda; 2004–2010. AIDS Research and Human Retroviruses 2012 Oct 9.: [Epub ahead of print]. 10.1089/AID.2012.0170 23046049

[8] Kiwanuka N, Robb M, Laeyendecker O, Kigozi G, Wabwire-Mangen F, et al (2010) HIV-1 viral subtype differences in the rate of CD4+ T-cell decline among HIV seroincident antiretroviral naive persons in Rakai district, Uganda. J Acquir Immune Defic Syndr 54: 180–184. 20010433 10.1097/QAI.0b013e3181c98fc0 PMC2877752

[9] Kaleebu P, Ross A, Morgan D, Yirrell D, Oram J, et al (2001) Relationship between HIV-1 Env subtypes A and D and disease progression in a rural Ugandan cohort. Aids 15: 293–299. 11273208 10.1097/00002030-200102160-00001

[10] Kiwanuka N, Laeyendecker O, Robb M, Kigozi G, Arroyo M, et al (2008) Effect of human immunodeficiency virus Type 1 (HIV-1) subtype on disease progression in persons from Rakai, Uganda, with incident HIV-1 infection. J Infect Dis 197: 707–713. 18266607 10.1086/527416

[11] Lutalo T, Gray RH, Wawer M, Sewankambo N, Serwadda D, et al (2007) Survival of HIV-infected treatment-naive individuals with documented dates of seroconversion in Rakai, Uganda. AIDS 21 Suppl 6S15–19. 18032934 10.1097/01.aids.0000299406.44775.de

[12] Kaleebu P, French N, Mahe C, Yirrell D, Watera C, et al (2002) Effect of human immunodeficiency virus (HIV) type 1 envelope subtypes A and D on disease progression in a large cohort of HIV-1-positive persons in Uganda. J Infect Dis 185: 1244–1250. 12001041 10.1086/340130

[13] Vasan A, Renjifo B, Hertzmark E, Chaplin B, Msamanga G, et al (2006) Different rates of disease progression of HIV type 1 infection in Tanzania based on infecting subtype. Clin Infect Dis 42: 843–852. 16477563 10.1086/499952

[14] Kaleebu P, Nankya IL, Yirrell DL, Shafer LA, Kyosiimire-Lugemwa J, et al (2007) Relation between chemokine receptor use, disease stage, and HIV-1 subtypes A and D: results from a rural Ugandan cohort. J Acquir Immune Defic Syndr 45: 28–33. 17310935 10.1097/QAI.0b013e3180385aa0

[15] Hoffman TL, Doms RW (1998) Chemokines and coreceptors in HIV/SIV-host interactions. AIDS 12 Suppl A: S17–26. 9632980

[16] Tscherning C, Alaeus A, Fredriksson R, Bjorndal A, Deng H, et al (1998) Differences in chemokine coreceptor usage between genetic subtypes of HIV-1. Virology 241: 181–188. 9499793 10.1006/viro.1997.8980

[17] Alaeus A, Lidman K, Bjorkman A, Giesecke J, Albert J (1999) Similar rate of disease progression among individuals infected with HIV-1 genetic subtypes A-D. AIDS 13: 901–907. 10371170 10.1097/00002030-199905280-00005

[18] Gray RR, Tatem AJ, Lamers S, Hou W, Laeyendecker O, et al (2009) Spatial phylodynamics of HIV-1 epidemic emergence in east Africa. AIDS 23: F9–F17. 19644346 10.1097/QAD.0b013e32832faf61 PMC2742553

[19] Sacktor N, Nakasujja N, Skolasky RL, Rezapour M, Robertson K, et al (2009) HIV subtype D is associated with dementia, compared with subtype A, in immunosuppressed individuals at risk of cognitive impairment in Kampala, Uganda. Clin Infect Dis 49: 780–786. 19622045 10.1086/605284 PMC2941149

[20] Easterbrook PJ, Smith M, Mullen J, O’Shea S, Chrystie I, et al (2010) Impact of HIV-1 viral subtype on disease progression and response to antiretroviral therapy. J Int AIDS Soc 13: 4. 20205896 10.1186/1758-2652-13-4 PMC2827379

[21] Carr JK (2006) Viral diversity as a challenge to HIV-1 vaccine development. Curr Opin HIV AIDS 1: 294–300. 19372824 10.1097/01.COH.0000232344.23533.be

[22] Hemelaar J, Gouws E, Ghys PD, Osmanov S (2011) Global trends in molecular epidemiology of HIV-1 during 2000–2007. AIDS 25: 679–689. 21297424 10.1097/QAD.0b013e328342ff93 PMC3755761

[23] Taylor BS, Sobieszczyk ME, McCutchan FE, Hammer SM (2008) The challenge of HIV-1 subtype diversity. New England Journal of Medicine 358: 1590–1602. 18403767 10.1056/NEJMra0706737 PMC2614444

[24] Morgan D, Malamba SS, Maude GH, Okongo MJ, Wagner HU, et al (1997) An HIV-1 natural history cohort and survival times in rural Uganda. AIDS 11: 633–640. 9108945 10.1097/00002030-199705000-00011

[25] Mulder DW, Nunn AJ, Wagner HU, Kamali A, Kengeya-Kayondo JF (1994) HIV-1 incidence and HIV-1-associated mortality in a rural Ugandan population cohort. AIDS 8: 87–92. 8011241 10.1097/00002030-199401000-00013

[26] Mbulaiteye SM, Mahe C, Ruberantwari A, Whitworth JA (2002) Generalizability of population-based studies on AIDS: a comparison of newly and continuously surveyed villages in rural southwest Uganda. Int J Epidemiol 31: 961–967. 12435768 10.1093/ije/31.5.961

[27] Ministry of Health, Republic of Uganda (2003) National Antiretroviral Treatment and Care Guidelines for Adults and Children. First edition ed: Kampala.

[28] Ministry of Health, Republic of Uganda (2008) National Antiretroviral Treatment and Care Guidelines for Adults and Children. Second ed: Kampala.

[29] Kaleebu P, Whitworth J, Hamilton L, Rutebemberwa A, Lyagoba F, et al (2000) Molecular epidemiology of HIV type 1 in a rural community in southwest Uganda. AIDS Res Hum Retroviruses 16: 393–401. 10772525 10.1089/088922200309052

[30] Yirrell DL, Kaleebu P, Morgan D, Watera C, Magambo B, et al (2002) Inter- and intra-genic intersubtype HIV-1 recombination in rural and semi-urban Uganda. AIDS 16: 279–286. 11807313 10.1097/00002030-200201250-00018

[31] Ssemwanga D, Ndembi N, Lyagoba F, Bukenya J, Seeley J, et al (2012) HIV type 1 subtype distribution, multiple infections, sexual networks, and partnership histories in female sex workers in Kampala, Uganda. AIDS Res Hum Retroviruses 28: 357–365. 21749285 10.1089/aid.2011.0024

[32] Ssemwanga D, Lyagoba F, Ndembi N, Mayanja BN, Larke N, et al (2011) Multiple HIV-1 infections with evidence of recombination in heterosexual partnerships in a low risk Rural Clinical Cohort in Uganda. Virology 411: 113–131. 21239033 10.1016/j.virol.2010.12.025 PMC3041926

[33] Gottlieb GS, Nickle DC, Jensen MA, Wong KG, Grobler J, et al (2004) Dual HIV-1 infection associated with rapid disease progression. Lancet 363: 619–622. 14987889 10.1016/S0140-6736(04)15596-7

[34] Alaeus A (2000) Significance of HIV-1 genetic subtypes. Scand J Infect Dis 32: 455–463. 11055646 10.1080/003655400458695

[35] Pant Pai N, Shivkumar S, Cajas JM (2012) Does genetic diversity of HIV-1 non-B subtypes differentially impact disease progression in treatment-naive HIV-1-infected individuals? A systematic review of evidence: 1996–2010. J Acquir Immune Defic Syndr 59: 382–388. 22269800 10.1097/QAI.0b013e31824a0628

[36] Kanki PJ, Hamel DJ, Sankale JL, Hsieh C, Thior I, et al (1999) Human immunodeficiency virus type 1 subtypes differ in disease progression. J Infect Dis 179: 68–73. 9841824 10.1086/314557

[37] Huang W, Eshleman SH, Toma J, Fransen S, Stawiski E, et al (2007) Coreceptor tropism in human immunodeficiency virus type 1 subtype D: high prevalence of CXCR4 tropism and heterogeneous composition of viral populations. J Virol 81: 7885–7893. 17507467 10.1128/JVI.00218-07 PMC1951291

[38] Wright E, Mugaba S, Grant P, Parkes-Ratanshi R, Van der Paal L, et al (2011) Coreceptor and cytokine concentrations may not explain differences in disease progression observed in HIV-1 clade A and D infected Ugandans. PLoS One 6: e19902. 21655330 10.1371/journal.pone.0019902 PMC3104992

[39] Dilys Morgan CM, Billy Mayanja, Martin Okongo, Rosemary Lubega, James Whitworth (2002) HIV-1 infection in rural Africa: is there a difference in median time to AIDS and survival compared with that in industrialized countries? AIDS 16: 597–603. 11873003 10.1097/00002030-200203080-00011

[40] Kitchen M, Quigley MA, Mwinga AM, Fuchs D, Lisse IM, et al (2008) HIV progression and predictors of mortality in a community-based cohort of Zambian adults. Journal of the International Association of Physicians in AIDS Care (JIAPAC) 7: 17–26. 17989427 10.1177/1545109707303989

[41] Metallidis S TO, Skoura L, Zebekakis P, Chrysanthidis T, et al.. (2013) Older HIV-infected patients-an underestimated population in northern Greece: epidemiology, risk of disease progression and death. Int J Infect Dis 2013 Apr 29: pii: S1201–9712(1213)00116–00111. doi: 00110.01016/j.ijid.02013.00102.00023. [Epub ahead of print]. 10.1016/j.ijid.2013.02.023 23639484

[42] WHO/UNAIDS/UNICEF Global HIV/AIDS response - Epidemic update and health sector progress towards Universal Access. Progress Report 2011. WHO Geneva, 2011.

[43] Farzadegan H, Hoover DR, Astemborski J, Lyles CM, Margolick JB, et al (1998) Sex differences in HIV-1 viral load and progression to AIDS. Lancet 352: 1510–1514. 9820299 10.1016/S0140-6736(98)02372-1

[44] Anastos K, Gange SJ, Lau B, Weiser B, Detels R, et al (2000) Association of race and gender with HIV-1 RNA levels and immunologic progression. J Acquir Immune Defic Syndr 24: 218–226. 10969345 10.1097/00126334-200007010-00004

[45] French N, Mujugira A, Nakiyingi J, Mulder D, Janoff EN, et al (1999) Immunologic and clinical stages in HIV-1-infected Ugandan adults are comparable and provide no evidence of rapid progression but poor survival with advanced disease. J Acquir Immune Defic Syndr 22: 509–516. 10961614 10.1097/00126334-199912150-00013

